# Molecular Diagnosis in Culture-Negative Meningococcal Arthritis: A Case Report and Case-Based Literature Review

**DOI:** 10.31138/mjr.160225.dcn

**Published:** 2025-12-31

**Authors:** Ruth Moreno-Soler, Maria Rico-Sánchez, Jorge Peris-García, Victoria Ortiz de la Tabla-Ducasse, Francisco Jover-Diaz

**Affiliations:** 1 University Miguel Hernández Alicante, Spain;; 2 Infectious Diseases Unit, Hospital Clínico San Juan de Alicante, Spain;; 3 Microbiology Department, Hospital Clínico San Juan de Alicante, Spain

**Keywords:** meningococcal arthritis, molecular diagnosis, culture negative

## Abstract

**Background:**

Meningococcal arthritis is a rare manifestation of invasive meningococcal disease, accounting for 1–12.5% of cases. Accurate diagnosis relies on synovial fluid analysis, blood cultures, and molecular techniques.

**Case Report:**

A 64-year-old male with a history of renal and rectal cancer presented with fever, lower limb oedema, and headache. Laboratory findings showed elevated inflammatory markers. Synovial fluid analysis was performed, and while cultures were negative, FilmArray Sepsis® Panel PCR was positive for *Neisseria meningitidis*, confirming the diagnosis of meningococcal septic arthritis.

**Discussion:**

A review of nine culture-negative meningococcal arthritis cases diagnosed using molecular methods is presented. Molecular diagnostic techniques, particularly PCR, offer significant advantages over traditional culture methods, including higher sensitivity and the ability to detect pathogens after antibiotic administration. In England and Wales, meningococcal septic arthritis represented 2% of all invasive meningococcal disease cases from 2010–2020. This case highlights the importance of molecular diagnostic techniques in accurately diagnosing meningococcal arthritis, especially when traditional culture methods fail. PCR-based methods are becoming essential tools in the management of this condition, potentially revealing a higher incidence than previously recognised.

## BACKGROUND

Meningococcal arthritis is a rare manifestation of invasive meningococcal disease caused by *Neisseria meningitidis*, accounting for approximately 1–12.5% of all meningococcal infections.^1^ It can present as either primary arthritis or secondary to meningococcal meningitis or septicaemia. The clinical presentation typically involves acute onset of joint pain and swelling, primarily affecting large joints such as knees and ankles.^2^ The highest incidence peak occurs around age 20, with additional peaks in early childhood and adults over 65 years old.^3^

Accurate diagnosis of meningococcal arthritis relies on a combination of synovial fluid analyses, blood cultures, and molecular techniques. Synovial fluid culture is positive in approximately 84% of cases, while blood cultures are positive in about 32.5% of cases.^2^ Polymerase chain reaction (PCR) of synovial fluid or blood samples has improved detection rates, especially in culture-negative cases.^4^ The use of advanced molecular techniques like the FilmArray® Sepsis panel has shown promise in detecting N. *meningitidis* in synovial fluid, potentially revealing a higher true incidence of meningococcal arthritis than previously recognised.^4^

## CASE REPORT

A 64-year-old male with a medical history of stage I left papillary renal carcinoma (status post-left nephrectomy in 2019), cT3N2M0 mid-rectal adenocarcinoma (diagnosed January 2022; treated with anterior resection in July 2022 and ileostomy closure in 2023), and pulmonary thromboembolism (March 2022) presented to the Emergency Department on September 29, 2024. The patient, who was unvaccinated per immunosuppression protocols, reported a 4-day history of fever (39.5°C), left lower limb oedema and pain, intense headache, and malaise. Initial evaluation revealed blood pressure of 168/88 mmHg, heart rate of 83 bpm, oxygen saturation of 98%, and temperature of 37.7°C. Laboratory findings included elevated inflammatory markers (CRP: 25.68 mg/dL; procalcitonin: 2.3 ng/mL; leucocytosis with 80.8% neutrophils) and acute kidney injury (urea: 74 mg/dL; creatinine: 2.33 mg/dL; eGFR: 28.38 mL/min).

During hospitalisation, petechial erythematous lesions developed on both tibias, with persistent left limb oedema. Imaging studies excluded deep vein thrombosis but revealed tibiotalar joint effusion. Empirical intravenous ceftriaxone and cefazolin were initiated after blood cultures were collected, in accordance with the recommended regimen for acute septic arthritis as outlined in the Guidelines for the Diagnosis and Treatment of Septic Arthritis in Adults and Children, developed by the GEIO (SEIMC), SEIP, and SECOT.^5^

Synovial fluid analysis (1.5 mL inflammatory-appearing fluid; no microcrystals) obtained three days post-admission showed negative cultures. Gram stain in synovial fluid was negative. Multiplex PCR (FilmArray® Sepsis Panel) of synovial fluid detected *Neisseria meningitidis*, confirming meningococcal septic arthritis. The patient received ceftriaxone (2g/12h IV) and low-dose prednisone (5mg/12h), with rapid clinical and biochemical improvement. After 7 days of hospitalisation, he was discharged on a 14-day ceftriaxone regimen and a prednisone taper.

Written informed consent was obtained from the patient for publication of this case report and any accompanying images.

## DISCUSSION

After searching PubMed, Scopus, and DOAJ databases using descriptors “meningococcal arthritis”, “culture negative”, “molecular diagnosis” we reviewed nine adult cases^6–14^ of culture-negative meningococcal arthritis diagnosed using molecular methods (**Table 1**). The patients range in age from 19 to 76 years old, with four males and three females. Most cases involve monoarthritis, primarily affecting the knee joint. Molecular diagnostic techniques were crucial, with PCR being the most common method used in 6 cases, while one case utilised ELISA. In a study on meningococcal septic arthritis (MSA) in England and Wales from 2010–2020, 162 cases were identified, representing 2% of all invasive meningococcal disease cases during the study period. Knee involvement was more frequent in adults, while hip involvement was more common in children and adolescents. MSA cases peaked in older adults (>45 years) and young children less than 5 years.^16^ In septic arthritis, when cultures are taken correctly, the microorganism is isolated in the synovial fluid in up to 80% of cases^17^ and in the blood by 25–35%.^18^

**Table 1.** Clinical and microbiological characteristics of primary meningococcal arthritis cases.

**Author/Year**	**Age/Sex**	**Clinical Presentation**	**Microbiological Cultures**	**Molecular Biology Tests (positive for** *N. meningitidis***)**	**Treatment Received**
Garner et al., 2011^6^	76/M	Fever, monoarticular (shoulder arthritis)	Negative blood and synovial fluid culture	Positive broad range 16S rDNA PCR	Ceftriaxone + arthroscopic drainage
Edgeworth et al, 1998^7^	24/M	Fever, monoarticular (knee arthritis)	Negative blood and synovial fluid culture	Positive PCR ELISA technique Positive 16S amplification	G Penicillin + arthroscopic drainage
Erviti et al, 2021^8^	41/M	Fever, cough, monoarticular (wrist) in COVID-19 infected patient	Negative blood and synovial fluid culture	by PCR and *Neisseria-*specific PCR (serogroup W135).	Intravenous ceftriaxone, 2 weeks.
Fox-Lewis et al., 2016^9^	52/M	Fever and rigors, maculopapular rash, monoarticular (knee). HIV positive patient.	Negative blood and synovial fluid culture	Positive PCR to detect broad-range bacterial 16S ribosomal DNA	Intravenous meropenem
Verma et al, 2015^10^	19/M	Polyarticular, purpuric rash (knee)	Diplococci gram negative in synovial Gram stain, negative blood and synovial fluid culture.	Positive PCR	G Penicillin + arthroscopic drainage
**Joyce et al., 2003^11^**	**19/F**	**Monoarticular (knee), fever, purpuric rash.**	**Diplococci gram negative in synovial Gram stain, negative blood and synovial fluid culture**	**Positive PCR**	**Intravenous benzylpenicillin (2 weeks) + arthroscopic drainage**
Petty et al., 1983^12^	23/M	A bacteriologist with polyarticular arthritis (elbow, knee)	Diplococci gram negative in synovial Gram stain, culture negative.	Enzyme-linked immunosorbent assay of synovial fluid	G Penicillin + Drainage
**Ahmed et al., 2021^13^**	**63/F**	**Sore throat, fever and rash on her legs. Painful, stiff and swollen knees.**	**No organisms identified on Gram Stain and no growth at 48 h on primary culture or at 7 days on extended culture**	**Positive synovial fluid for broad-based bacterial 16S rDNA PCR molecular testing**	**Co-amoxiclav**
**Noteman TW et al., 2020^14^**	**80/M**	**Fever, myalgia with worsening pain in his shoulders and right knee**	**High numbers of leukocytes and moderate numbers of Gram-positive cocci. There was no direct growth.**	**Positive synovial fluid for broad-based bacterial 16S rDNA PCR molecular testing**	**Benzylpenicillin (2.4 g, 6 hourly)**
Moreno-Soler et al., 2025 (present case)	64/M	Fever, monoarticular (knee arthritis)	Negative blood and synovial fluid culture	Positive PCR multiplex (FilmArray Sepsis® Panel)	Ceftriaxone 2 weeks

Given the patient’s symptoms (fever, severe headache, petechial rash), CNS infection could be considered as a differential diagnosis. However, lumbar puncture was not performed. Cerebrospinal fluid (CSF) PCR or culture for *N. meningitidis* could have further supported the diagnosis of invasive meningococcal disease. Occhi-Gómez et al.^1^ found that up to 16% of synovial cultures were negative, Also Schaad et al.^17^ reported between 10% to 20% of cases being culture negative. It’s important to note that while these percentages are high, they still leave a significant number of cases where the causative organism may not be identified through traditional culture methods.

Molecular diagnostic techniques have significantly improved the detection and identification of *Neisseria meningitidis* in cases of meningococcal arthritis, offering several advantages over traditional culture methods. Sacchi et al.^18^ prospectively examined CSF samples from patients with suspected meningitis using broad-range bacterial PCR targeting the 16S rRNA gene, followed by sequencing. *Neisseria meningitidis* was identified in several cases, including some that were culture-negative, demonstrating that 16S rDNA PCR can improve the etiologic diagnosis of bacterial meningitis, especially when conventional cultures are negative or when patients have received prior antibiotics. However, the authors note that Gram stain and culture remain essential, and PCR results should be interpreted in the clinical context to avoid false positives due to contamination or detection of non-viable bacteria. Molecular diagnosis can directly detect *N. meningitidis* DNA from synovial fluid samples. The sensitivity of PCR has been shown to be higher (47%) than blood cultures (31%) with high specificity (96%).^15^ We have not performed this technique representing a limitation to final diagnosis.

PCR results are not affected by prior antibiotic administration as the DNA can still be detected even if there are no viable bacteria to culture-hence making it a useful test in culture negative samples. Real-time PCR (rtPCR) assays can simultaneously detect multiple bacterial pathogens, including N. meningitidis, in a single reaction, as in our case (FilmArray® sepsis panel). Some studies have revealed that primary meningococcal arthritis may be more common than previously thought, as it can be easily misdiagnosed without specific testing.^7^

In conclusion, molecular diagnostic techniques, particularly PCR-based methods, have significantly improved the detection and identification of *N. meningitidis* in cases of meningococcal arthritis, offering advantages over traditional culture methods. The use of advanced molecular techniques like FilmArray® Sepsis panel could be potentially promising in detecting *N. meningitidis* in synovial fluid, theoretically revealing a higher true incidence of meningococcal arthritis than previously recognised.
